# Unveiling the influence of the Italian mafia as a Dark Triad threat on individuals’ affective states and the power of defense mechanisms

**DOI:** 10.1038/s41598-023-38597-6

**Published:** 2023-07-25

**Authors:** Sandra J. Diller, Janna Hämpke, Gianluca Lo Coco, Eva Jonas

**Affiliations:** 1 Seeburg Castle University, Seekirchen am Wallersee, Austria; 2 LMU Center for Leadership and People Management, Munich, Germany; 3 LMU Munich, Munich, Germany; 4 Università degli Studi di Palermo, Palermo, Italy; 5 University of Salzburg, Salzburg, Austria

**Keywords:** Psychology, Human behaviour

## Abstract

The present research investigated whether the Italian mafia as a Dark Triad threat increased threat-related affective states and explored how thinking about defense mechanisms may help to reduce these states. For this, we conducted a multi-method experimental study with Italians (*N* = 253). The quantitative results show that the mafia as a threat manipulation increased threat-related affective states in terms of higher behavioral inhibition (BIS) and lower behavioral activation (BAS). The qualitative results further depict proximal and distal defense mechanisms to reduce this threat, which can be categorized into models of threat and defense. Exploratory analyses indicate that naming distal defenses positively affected the increase of BAS. Additionally, when participants had higher levels of BIS after the threat, naming more defenses and proximal defenses positively affected the decrease of BIS. Further qualitative results provide valuable information on effective personal and societal buffers for the perceived threat of the Italian mafia.

## Introduction

The Italian mafia has gained significant notoriety as one of the prominent criminal organizations in Europe. The term “mafia” is hereby applied to groups and individuals who wanted to gain power over political and economic events in their city by threatening or using violence^1^, becoming a synonyme for organized crime^2^. The Italian mafia includes the *Cosa Nostra* in the Western part of Sicily, the *Ndrangheta* from Calabria, the *Camorra* from Naples^1,3^, and the *Apulian Mafia*^4,5^. While these four organizations differ in terms of their history, symbolism, rituals, criminal interests, organizational structure, and modus operandi^6,7^, they show similarities in their ongoing violent strive for power^3,5^ for which they are frightened by the Italian population (e.g.^8,9^). However, research on the Italian mafia as a threat and how the population deals with the threat of the mafia to reduce their negative affective states is rare has not been researched. By investigating the mafia as Dark Triad (DT) threat, the present multi-method study gives valuable insights into people’s threat reaction to the mafia and their use of defense mechanisms to deal with the threat.

Over the last decades, the Italian mafia has constantly exercised power to influence both the economic and political systems. For instance, it actively intervenes with violence to gain control over territories and further expand its political influence^1,3,10,11^. Such political violence is assumed to be connected to assassinations of and attacks on Italian politicians that significantly increase before and after elections^10,12^. In addition to controlling political decisions, the mafia also uses its power to control illicit trade, claim ill-gotten gains from economic actors, and control private economic activities^13^. The aim of infiltrating the economic system is to maximize their profit with rent extraction or laundering money. The large profits from illegal activities are invested in both illegal markets and the legal economy, which in turn allows the mafia to expand their political rule by penetrating and controlling legal sectors of the economy^1,3^. To gain control over a given territory and thus increase their political power in that area, as well as to establish contacts with legal entrepreneurs and penetrate the legal economy, the Italian mafia further does not recoil from techniques of extortion, such as demanding money or materials in exchange for protection^1,3,14,15^.

By using violence, attacks, or different techniques of assertion the Italian mafia has become famous for its ruthless and reckless behavior. Quotes attributed to (former) Italian mafia members further illustrate the mafia’s willingness to use force, such as “You can get much further with a kind word and a gun than you can with a kind word alone” (Al Capone) and “I can’t stand squealers, hit that guy!” (Albert Anastasia).

Mafia members therefore resort to violence, murder, and/or other coercive and unethical means - a behavior that makes them rise in the command hierarchy or maintain their position of power^16^. This kind of behavior, such as relational devaluation (e.g., power games, megalomania, or displacement of guilt), can be threatening to one’s basic needs, emotional well-being, and physical health^17,18^. Furthermore, such ruthless and reckless behavior to gain power can also be perceived as a Dark Triad threat^19^.

The Dark Triad (DT) is a cluster of the three personality traits (narcissism, Machiavellianism, and psychopathy) that all share a decreased level of morality, agreeableness, and social emotions^20–22^, as well as a high strive for power^21,23^. The DT is further related to abusive supervision, counterproductive work behavior, and bullying in the organizational context^24–28^, showing detrimental effects on subordinates and the organization^29–41^. Previous research shows that the mafia is connected to the DT: Imprisoned mafia members scored higher in psychopathy than non-mafioso imprisoners arrested for a similar crime^42^ and previous research has connected the mafia to narcissism^43–46^.

Due to its destructive and demoralized behavior, the DT can be perceived as a possible threat, leading to their victims’ alertness, anxiety, avoidance, and inhibition^19^. This change in affective states can be explained by the Reinforcement Sensitivity Theory: According to the General Process Model of Threat and Defense by Jonas et al.^47^, when people are confronted with an anticipated threat (e.g., something that might be coming and could harm you), they want to avoid it and are conflicted with what to do, leading to high vigilance, avoidance, and/or arousal. Thus, people confronted with an anticipated threat respond with a higher activation of the behavioral inhibition system (BIS) (e.g., higher affective states of inhibition and anxiety) and a lower activation of the behavioral approach system (BAS) (e.g., lower affective states of goal-orientation and relaxation)^47,48^. This process differs from people being confronted with an *actual* threat (e.g., the lion in front of me): Then the Fight-Flight-Freeze system (FFFS) sets on, leading to either *flying* out of the threating situation, *fighting* against the threat, or *freezing*/*frighting/fainting* as a kind of staying in an inhibited, hyper-vigilant, and hyper-anxious state^49^. The mafia as an *anticipated* DT threat (the possibility of having contact with the Italian mafia) could therefore lead to higher BIS (anxiety, inhibition, and avoidance) and lower BAS (goal-orientation, relaxation) and as an *actual* threat (the mafia boss in front of me) lead to fight/flight/freeze/fright/faint.

**Figure 1:**
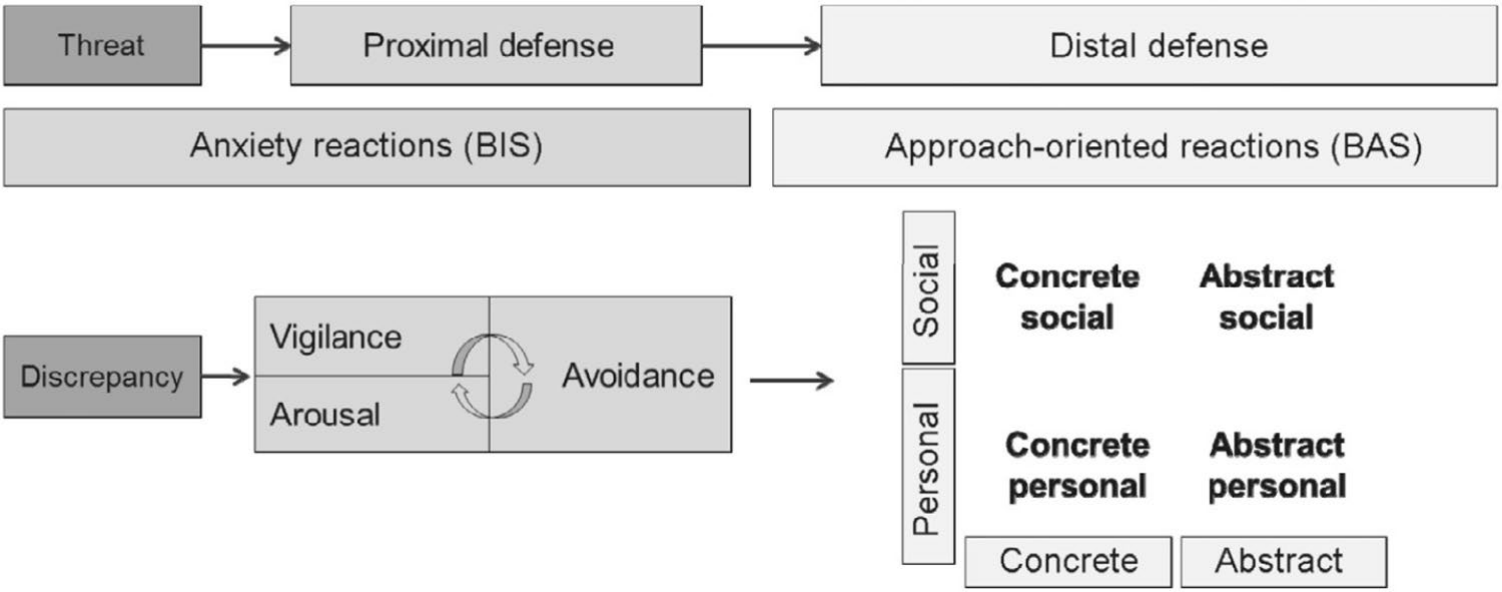
General process model of threat and defense by Jonas et al.^47^.

If BIS activation can be decreased or BAS activation can be increased, the BAS instead of the BIS sets on, helping with concrete or abstract solutions. This BAS activation leads to different distal defense mechanisms that can either directly deal with the threat (concrete distal defenses), such as by doing something against the mafia, or it can indirectly deal with the threat (abstract distal defenses), such as thinking about better times. Such abstract and concrete distal defenses cannot only be on a personal level as the examples showed but also on a social level: In other words, regarding a concrete defense, other people, such as family members or institutions, can be involved in the defense, or regarding an abstract defense, shared worldviews can be involved^47,48^. Figure 1 summarizes the complete process from threat to defense with its various forms of defense mechanisms.

To summarize, the mafia can be a DT threat, which could lead to higher BIS and lower BAS activation as well as proximal and distal defense mechanisms. To explore this assumption, the present research investigated whether the mafia as a DT threat leads to higher BIS and lower BAS activation, and how people deal with this threat when it comes to defense mechanisms. As the mafia could be considered as DT (e.g.^45^) we used the Italian mafia as a DT manipulation controlled by a DT manipulation check. Given that the DT can be perceived as a threat, increasing BIS and lowering BAS (e.g.^19^), we further hypothesized that this manipulation leads to this respective change in affective states. As thinking about defense mechanisms after a threat should again decrease BIS and increase BAS (e.g.^50^), we further expected a shift back. Thus, our first research question concerns the measured change in BIS and BAS after the mafia DT threat (Research question 1), concluding with the following two hypotheses:

### H1a

BIS increased after the mafia threat and decreased after thinking about the defense mechanisms.

### H1b

BAS decreased after the mafia threat and increased after thinking about the defense mechanisms.

In a second research question, we further wanted to explore which defense mechanisms help best when confronted with a mafia DT threat (Research question 2). For this question, we inductively qualitatively analyzed the defense mechanisms people used after being confronted with the mafia DT threat as well as categorized them into the General Process Model of Threat and Defense^47^ as a first step. As a second step, we explored their relationship to the BIS and BAS affective states after the threat versus after the defense mechanisms to investigate the change of BIS and BAS dependent on the defense mechanisms named.

Lastly, a third research question qualitatively conducted possible buffers for such a threat situation. In other words, we investigated the societal and personal resources that could help to buffer the DT threat of the Italian mafia (Research question 3), as the process from threat to defense can be buffered by the situational and personal context^47^. This multi-method research approach gives valuable insights into how people react to the mafia perceived as a DT threat and which defense mechanisms can be used to effectively reduce this perceived threat.

## Results

### Manipulation check

To check whether the mafia was perceived as a DT threat in our sample, we analyzed both the DT statements and the open answer fields (see “Methods” section). Concerning the DT statements, an average of 5.65 (*SD* = 2.67) of the 12 statements were clicked on. The inductive qualitative data analysis revealed that the Italian mafia is perceived to be present in Italy (*n* = 236), although it was described by some participants to be less visible now than in the past (*n* = 35). Especially the mafia’s demand for protection money and threats were often mentioned by the participants (*n* = 196) but also its infiltration into the social and political system (*n* = 79), negatively affecting the Italian economy and society (e.g., less economic growth) (*n* = 82) and leading to a corrupt report system (*n* = 38). This made participants feel scared (*n* = 172), angry (*n* = 86), helpless (*n* = 82), inhibited (*n* = 51), alone (*n* = 19), and frustrated (*n* = 46). Furthermore, the Italian mafia was described as a criminal and deadly organization (*n* = 49). Hence, considering both the quantitative and qualitative results the manipulation of this study worked well and the mafia was perceived as DT threat.

### Research question 1

**Figure 2:**
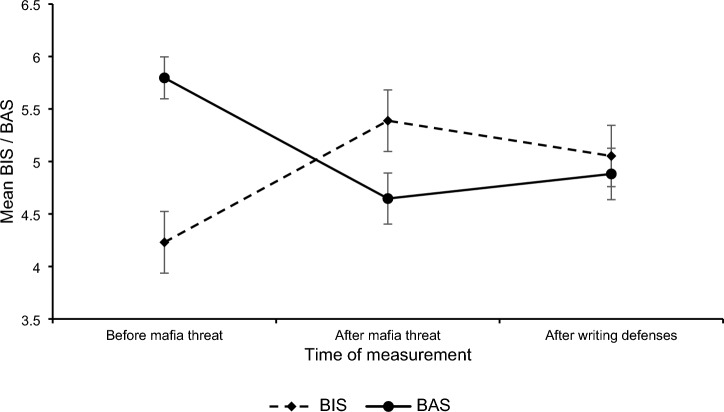
BIS and BAS change before the threat, after the threat, and after the defense. *BIS* behavioral inhibition system, *BAS* behavioral activation system.

Two repeated measures ANOVAs with a Greenhouse–Geisser correction showed that BIS levels, *F*(1.46, 366.91) = 54.93, *p* < 0.001, *η*^2^ = 0.18, as well as BAS levels, differed between the measures (before the threat, after the threat, and after the defenses), *F*(1.45, 364.98) = 77.51, *p* < 0.001, *η*^2^ = 0.24. As depicted in Fig. 2 and in line with *H1a* and *H1b*, BIS increased, *F*(1, 251) = 81.38, *p* < 0.001, *η*^2^ = 0.25, *M*_*before Threat*_ = 4.22 (*SD* = 2.38), *M*_*after Threat*_ = 5.38 (*SD* = 2.37), and BAS decreased after threat, *F*(1, 251) = 104.50, *p* < 0.001, *η*^2^ = 0.29, *M*_*before Threat*_ = 5.80 (*SD* = 1.61), *M*_*after Threat*_ = 4.65 (*SD* = 1.96). In line with *H1a* and *H1b*, after the defenses BIS decreased, *F*(1, 251) = 22.37, *p* < 0.001, *η*^2^ = 0.08, *M*_*after Defenses*_ = 5.04 (*SD* = 2.36), and BAS increased, *F*(1, 251) = 15.23, *p* < 0.001, *η*^2^ = 0.06, *M*_*after Defenses*_ = 4.88 (*SD* = 1.98).

### Research question 2

#### Qualitative inductive analysis

At first, the defense mechanisms were qualitatively analyzed and categorized into the General Process Model of Threat and Defense^47^. As depicted in Table 1, participants named several proximal and distal defense mechanisms with the most named being report/denounce them (*n* = 105; #23: “I would denounce it whatever it takes”), be afraid (n = 36; #7: “I would be afraid for my safety and the safety of my family members”), and give in/acquiesce everything (n = 36; #132: “I would try to acquiesce to their requests”). Based on the responses, which included a variety of fight, flight, and freeze/fright/faint strategies, we decided to further categorize them into the FFFS as part of the Reinforcement Sensitivity Theory^49^, although the FFFS should only be triggered in an *actual* threat situation involving fear^51^. By categorizing the responses into the FFFS, we were able to discover an additional defense mechanism that can neither be described as fight, flight, or freeze/fright/faint. We have described it as ‘giving in’ as succumbing to the demands of the mafia (*n* = 31).

**Table 1:** Proximal and distal defense mechanisms mentioned.

	Freeze/fright/faint (*n* = 96)	Fight (*n* = 201)	Flight (*n* = 35)	Give in (*n* = 41)
Proximal defense (*n* = 96)				
Arousal (*n* = 40)	Be afraid (36), feel inhibited (8), keep calm (5), feel helpless (3), feel humiliated (1), be confused (1), negative affective states besides anxious inhibition (uncomfortable, sad, …) (1), trust my instincts (1)			
Avoidance (55)	I don’t know (31), do nothing (7), avoid contact (not communicate with them or others) (4), pretend nothing happened (2)			
Vigilance (*n* = 1)	Not make things worse (1), fail out of fear (1)			
Distal defense (*n* = 276)				
Concrete personal (*n* = 233)		Report/denounce them (105), not succumb/not accept this (13), fight back/rebel (11), think about a solution (10), take precautions (5), negotiate (4), stall them (4), not be silent (1), act decisively (1), gain information on them (1), be angry (1), make a will (1), take my own life (1)	Flight (give up business, change city/country, submerge) (34), dream about good times (1)	Give in/acquiesce everything (36), not report, take the deal (2), do anything to save the family (2), play their game (1)
Concrete social (*n* = 43)		Ask for help (e.g., by associations, institutions, offices) (28), talk to close ones (15)		

Numbers behind the categorization symbolize the number of participants that named this category.

#### Mixed-method-approach

As shown in Table 2, similarly to BIS and BAS (see “Methods” section), the number of qualitative answers for distal defenses and proximal defenses negatively correlated (*r* =  − 0.43, *p* < 0.001). Furthermore, BIS (quantitative measure) was associated positively with the number of qualitative answers for proximal defenses and negatively with the number of qualitative answers for distal defenses, with a significant association between BIS_after Threat_ and naming proximal defenses. Similarly, BAS was positively correlated with naming *distal defenses* and negatively with naming *proximal defenses*, with a significant correlation between naming *distal defenses* and BAS_after Defenses_.

**Table 2:** Correlations of the number of distal and proximal defenses with BIS and BAS measures after threat and after the defenses.

	BIS_*after Threat*_	BIS_*after Defenses*_	BAS_*after Threat*_	BAS_*after Defenses*_
*N*_*proximal defenses*_	0.16* (0.011)	0.10 (0.110)	− 0.09 (0.166)	− 0.10 (0.123)
*N*_*distal defenses*_	− 0.11 (0.076)	− 0.12 (0.063)	0.11 (0.079)	0.17** (0.008)

*p*-values are presented in parentheses.

*p < 0.05, **p < 0.01.

Significant vales are in bold.

To exploratively investigate what kind of defense mechanisms might have influenced the change in BIS and BAS after the threat to after writing defenses, we ran a series of regression analyses using different defense mechanisms as predictors and the *z*-standardized BIS_after threat_ or BAS_after threat_ as covariates. As presented in Table 3, the results show that naming *distal defenses* and, in particular, *personal distal defenses* had a significant positive effect on BAS_after threat_. In other words, the more *distal defenses* and, in particular, *personal distal defenses* were named, the higher the increase in BAS. The other defense mechanisms did not significantly influence the changes in BIS or BAS.

**Table 3:** Moderated regression analyses to BIS or BAS after defenses.

Model	BIS_after Defense_				BAS_after Defense_			
	Effect	*b*	SE *b*	*p*	Effect	*b*	SE *b*	*p*
1	Sum defenses	− 0.14	0.10	0.160	Sum defenses	0.13	0.08	0.123
	BIS_after Threat_	2.46***	0.16	< 0.001	BAS_after Threat_	1.90***	0.14	< 0.001
	**Interaction**	− 0.26*	**0.10**	**0.013**	Interaction	− 0.10	0.09	0.229
2	Proximal defenses	− 0.06	0.12	0.634	Proximal defenses	− 0.06	0.10	0.559
	BIS_after Threat_	2.19***	0.08	< 0.001	BAS_after Threat_	1.74***	0.07	< 0.001
	**Interaction**	− 0.25*	**0.12**	**0.032**	Interaction	0.02	0.10	0.807
3	Arousal	− 0.01	0.15	0.934	Arousal	− 0.02	0.12	0.854
	BIS_after Threat_	2.19***	0.07	< 0.001	BAS_after Threat_	1.74***	0.07	< 0.001
	**Interaction**	− 0.47**	**0.15**	**0.002**	Interaction	0.03	0.12	0.841
4	Avoidance	− 0.14	0.19	0.464	Avoidance	− 0.14	0.16	0.401
	BIS_after Threat_	2.09***	0.08	< 0.001	BAS_after Threat_	1.74***	0.06	< 0.001
	Interaction	0.02	0.19	0.901	Interaction	0.03	0.17	0.870
5	Vigilance	− 0.24	1.19	0.842	Vigilance	− 0.05	0.69	0.945
	BIS_after Threat_	2.09***	0.07	< 0.001	BAS_after Threat_	1.75***	0.06	< 0.001
	Interaction	1.86	2.46	0.450	Interaction	0.67	1.21	0.579
6	Distal defenses	− 0.06	0.10	0.559	**Distal defenses**	0.21*	**0.09**	**0.018**
	BIS_after Threat_	2.12***	0.13	< 0.001	BAS_after Threat_	1.90***	0.11	< 0.001
	Interaction	− 0.03	0.11	0.748	Interaction	− 0.14	0.08	0.086
7	Personal defenses	− 0.09	0.10	0.347	**Personal defenses**	0.25**	**0.08**	**0.003**
	BIS_after Threat_	2.12***	0.12	< 0.001	BAS_after Threat_	1.86***	0.10	< .001
	Interaction	− 0.03	0.10	0.753	Interaction	**0.14**	0.08	0.088
8	Social defenses	0.11	0.18	0.518	Social defenses	− 0.15	0.15	0.318
	BIS_after Threat_	2.09***	0.08	< 0.001	BAS_after Threat_	1.76***	0.06	< 0.001
	Interaction	0.03	0.20	0.869	Interaction	− 0.07	0.14	0.641
9	Freeze/fright/faint	− 0.06	0.12	0.634	Freeze/fright/faint	− 0.06	0.10	0.559
	BIS_after Threat_	2.19***	0.08	< 0.001	BAS_after Threat_	1.74***	0.07	< 0.001
	**Interaction**	− 0.25*	**0.12**	**0.032**	Interaction	0.02	0.10	0.807
10	Fight	− 0.09	0.10	0.396	Fight	0.14	0.09	0.111
	BIS_after Threat_	2.13***	0.11	< 0.001	BAS_after Threat_	1.76***	0.10	< 0.001
	Interaction	− 0.07	0.11	0.525	Interaction	− 0.04	0.09	0.630
11	Flight	− 0.06	0.20	0.779	Flight	− 0.18	0.18	0.319
	BIS_after Threat_	2.07***	0.08	< 0.001	BAS_after Threat_	1.73***	0.07	< 0.001
	Interaction	0.18	0.20	0.368	Interaction	0.07	0.17	0.661
12	Give in	0.12	0.19	0.545	Give in	0.28	0.16	0.079
	BIS_after Threat_	2.10***	0.07	< 0.001	BAS_after Threat_	1.82***	0.06	< 0.001
	Interaction	− 0.14	0.22	0.531	**Interaction**	− 0.41*	**0.17**	**0.015**

Unstandardized regression weights for significant interactions are presented. BIS_after threat_ or BAS_after threat_ are *z*-standardized. The *p*-value indicates an overall significant interaction effect.

*p < 0.05, **p < 0.01, ***p < 0.001.

Significant vales are in bold.

Based on a repeated measures model approach according to Wan^52^, we further examined the moderating role of BIS_after threat_ or BAS_after threat_ in the relationship between the defense mechanisms named and BIS_after defense_ or BAS_after defense_. As shown in Table 4, BIS_after threat_ moderated the effect of the *sum of defenses*, *proximal defenses*, *arousal*, and *freeze/fright/faint* on BIS_after defense_, meaning that participants with higher levels of BIS_after threat_ in comparison to those with average or lower levels experienced a significant decline in BIS from after the threat to after writing defenses when they named more defenses and, in particular, more proximal defenses. Additionally, we found that BAS_after threat_ moderated the relationship between *give-in* and BAS_after defense_, meaning that participants with lower levels of BAS_after threat_ experienced a stronger increase in BAS from after the threat to after writing defenses when they named more of giving-in defenses.

**Table 4:** Moderated associations between different defense mechanisms and BIS or BAS after threat.

Dependent variable	Defense mechanism	Moderator	Slope at − 1*SD*	Slope at *M*	Slope at + 1*SD*	*p*
BIS_after defense_	Sum defenses	BIS_after threat_	0.12	− 0.14	− 0.40**	0.013
	Proximal defenses	BIS_after threat_	0.20	− 0.06	− 0.31*	0.032
	Arousal	BIS_after threat_	0.46	− 0.01	− 0.49**	0.002
	Freeze/fright/faint	BIS_after threat_	0.20	− 0.06	− 0.31*	0.032
BAS_after defense_	Give in	BAS_after threat_	0.69***	0.28	− 0.12	0.019

Unstandardized regression weights for significant interactions are presented. BIS_after threat_ or BAS_after threat_ are *z*-standardized. The *p*-value indicates an overall significant interaction effect.

*p < 0.05, **p < 0.01, ***p < 0.001.

### Research question 3

#### Qualitative inductive analysis

As the process from threat to defense can be buffered by the situational and personal context^47^, we further qualitatively inductively investigated which societal and personal resources could help to buffer the DT threat of the Italian mafia. About half of the participants expressed trust in the police and suggested reporting the threat (*n* = 131; #199: “go to the authorities and report the threat”), while others wished for more effective law enforcement and legality in politics (*n* = 44; #193: “More assistance and real protection by law enforcement”; #10: “Actively monitor these individuals who are dangerous and harmful to society”; #42: “Even in law enforcement there are individuals who cooperate with the Mafia”; #248: “Politics made by righteous people who believe in legality”; #224: “The state doesn't always guarantee your protection”). Participants further mentioned the important role of information (*n* = 41; #4: “Raise awareness with social media about these issues”; #8: “Talk about what the Mafia is and how to fight it”; #27: “Raise awareness in the community about how shameful, as well as illegal, it is to collaborate with the mafia”; #234: “Educating the younger generation”; #189: “Spread the word among young people, make the younger ones open their eyes with school events and other initiatives”). In addition, participants called people to support social movements against the mafia, such as Addio Pizzo or Libera (*n* = 56; #125: “Join a movement to fight it together”; #152: “Join the Addio Pizzo movement”; #219: “Support associations like Libera”). Moreover, they encouraged people to refuse to pay (*n* = 44; #20: “Everyone should resist”; #21: “Refuse to pay lace”) and ask for help (*n* = 5; #9/#114/#131: “Ask for help”). Contrariwise, a few people suggested flight (*n* = 39), such as “mov[ing] to another state” (#16/#62/#69/#94/#108/#115/#126/#129/#171/#192/#208/#215/#225). Further mentions included the importance of job creation to reduce poverty and create alternatives for joining the mafia (*n* = 5; #14: “Giving jobs to young people in exploited areas”), the importance of psychological support (*n* = 3; #209: “Social and psychological assistance”), and the importance to “stop idolizing the mafia” (#51).

## Discussion

The Italian mafia is known for its ruthless and reckless behavior, for which they are frightened by the Italian population^8,9^. However, research on how the population deals with the threat of the mafia is rare. Building on previous studies that showed a link between the mafia and DT tendencies (e.g.^42,45^), the present multi-method research explored (1) whether the mafia perceived as a DT threat is leading to higher BIS and lower BAS activation (quantitative approach), (2) how people deal with this threat when it comes to defense mechanisms as well as how this relates to BIS and BAS activation (mixed-method approach), and (3) what could be done on an individual and societal level to buffer the perceived threat (qualitative approach).

Concerning Research question 1, the results of this study showed that BIS increased and BAS decreased after the threat of the mafia as well as BIS decreased and BAS increased after thinking about the defense mechanisms. This result is in support of our hypotheses H1a and H1b as well as in line with previous threat research (e.g.^50^).

Concerning the qualitative part of Research question 2, the results of our investigations of the defense mechanisms were in line with the General Process Model of Threat and Defense^47^. Additionally, participants reported fight, flight, and freeze/fright/faint strategies that can be categorized into the FFFS^49^. While the FFFS should be only activated in *actual* but not *anticipated* threat situations^51,53^, there are two assumptions that can be discussed: Firstly, the mafia threat might have fueled both processes of anxiety and fear, as Italians might have perceived this threat so real and physically close—an assumption that can be supported by previous research on how threats can be perceived closer as they are^54^. Secondly, this finding fuels the ambivalent debate about how different these two processes really are^51,55^. Table 1 provides a cross-over design that depicts how both processes can be connectively qualitatively researched. Furthermore, the Reinforcement Sensitivity Theory Affects Questionnaire (RST-AQ) provides a quantitative option to combine both processes^51^.

Furthermore, one personal distal defense mechanism, i.e. “giving in”, was named that cannot be categorized into the FFFS^49^. One explanation of why the strategy of giving in was shown is that DT threats might lead to feelings of helplessness^19^, which is why this could be a sign for learned helplessness (e.g.^56^). Another explanation of this finding is the dual concern model that suggests five ways of how people deal with a conflict: (a) to compete, force and fight, (b) to compromise in terms of finding a deal or collaborate in terms of finding the best solution for both parties, (c) to avoid the conflict, and (d) to accommodate / give in. This last strategy of giving in is mainly used when there is a higher concern towards the other party than towards one’s own goals^57^—particularly when this party is powerful: “Accommodation often occurs when there is a power differential between the parties and the high power party is willing to use a forcing approach to obtain what he or she wants. Realizing that the situation is futile, the low power party accommodates to the high power party, limiting any damage to the relationship or the organization”^58^ (p. 72).

Regarding the mixed-method part of Research question 2, we firstly discovered a positive relationship between BIS and proximal defenses, as well as a negative relationship between BIS and distal defenses. Similarly, we observed a positive relationship between BAS and distal defenses, as well as a negative relationship between BAS and proximal defenses. This finding is in line with the General Process Model of Threat and Defense (see Fig. 1^47^). By taking a closer look, we further found that naming more distal defenses and, in particular, more personal distal defenses seemed to help increase BAS. This finding is in line with the model, as personal concrete distal defense strategies help regain “personal control or self-efficacy [as well as can] provide a vision of clear and decisive goal pursuit”^47^ (p. 247). In addition, we found that people with higher levels of BIS had success in decreasing their BIS when writing about more defenses in general, independently of what they wrote about. This underlines the importance to exhibit a variety of defense mechanisms when dealing with negative affective states (e.g.^59,60^). By looking more closely, also naming more proximal defenses reduced participants’ BIS. Thus, writing about their BIS affective states seemed to have helped them feel less anxiously inhibited. Following previous clinical and non-clinical research on the effects of the expression of negative affective states, it can be supposed that the writing has led to enhanced self-acceptance, fostered self-understanding, and reduced inhibition of negative feelings, which, in turn, goes along with reduced feelings of stress (e.g.^61,62^). Additionally, we found that writing on giving in has helped people who felt lower levels of BAS after threat to increase their approach-oriented affective state. Although affective states can influence motivations and conflict strategies^63^, it is unclear why such a strategy where one gives up their control in some sense (e.g., control theory) can help BAS to regenerate. However, when looking at concrete personal defenses, such defenses do not need to be effective to work and as “compulsive reactions may also conflict with own goals and values” (p. 247) as can be seen by drug use or greedy consumption after threat^47^. In other words, giving in might just be an easy strategy people in their first impulse strive towards.

In terms of possible societal and personal buffers (Research question 3), most people suggested going to authorities, while others criticized that more effective law enforcement and legality in politics are needed. This finding highlights the importance of trust in political actors to keep a political system stable, especially when citizens feel threatened (e.g.^64–66^). Further suggestions for buffers include sharing information about the Italian mafia with the public and supporting movements against the mafia. Previous and recent social movements, such as the Fridays For Future climate protest, have emphasized the valuable opportunities social movements offer by building powerful collectives, raising awareness for the threats, and motivating policy changes before the threatening circumstances even increase (e.g.^67–69^).

### Limitations

Although the present research provides valuable findings, there are some limitations to consider. One first limitation of this study is its use of an Italian study sample and its restricted focus on the Italian mafia. However, the Italian mafia’s activities are not limited to Italy^70–72^ and other mafias besides the Italian mafia exist (e.g., Russian mafia^73^). Thus, future studies should investigate other national or international contexts. A second limitation addresses the use of a scenario as manipulation, as affective states elicited by scenarios can differ from the affective states experienced in a field study design (e.g.^74^). For instance, a recent study by Baraldi et al.^8^ showed the associations between the mafia’s organized crime infiltration and women’s reduced participation in politics due to feelings of fear. A third limitation concerns our mixed-method analyses with our defense strategies; future research could focus on replicating our findings by measuring possible defense mechanisms quantitatively to better conclude the relationship of these defense mechanisms to the affective states. A fourth limitation addresses the use of a dichotomous scale for the DT manipulation check, as a Likert scale could better quantify the perception of the mafia as a DT threat.

### Conclusion

In conclusion, the Italian mafia as a DT threat and their effect on the Italian population cannot be denied: Not only our results but also the first field studies on the Italian mafia (e.g.^8,9^) highlight the feelings of anxious inhibition and fear triggered by the Italian mafia. However, our results depict the importance of writing about possible defense mechanisms to reduce these threat-related affective states. One practical implication could therefore be to not only inform about the mafia but also inform about how important it is to think about various defense mechanisms. Teaching citizens to use concrete personal defenses might be especially important to increase their feelings of BAS. Furthermore, our results provided valuable starting points for societal interventions to buffer the process from threat to defense.

## Methods

### Sample

To calculate the sample size required to explore changes in BIS and BAS over our study, we conducted an a priori power analyses using G*Power^75^. Because of missing previous research on the mafia as DT threat, only approximation could be made. To detect a small effect size of *f* = 0.10 in a repeated measures ANOVA with a power of 0.80, α = 0.05, 3 points of measurements with a correlation of *r* = 0.50 among the measures, and a nonsphericity correction of ϵ = 1, at least *N* = 163 participants were needed.

The sample of this study consisted of 252 Italian people (41% male, 59% female) who were between 18 and 59 years old (*M* = 38.22, *SD* = 12.79). Supplementary Appendix 1 and 2 show further demographics of the sample. Of this sample, 10% reported having friends or family members involved with the mafia, as well as 2% reporting to have themselves been hurt by the mafia, 2% having friends or family hurt by the mafia, and 1% having friends or family killed by the mafia. Other 8% reported not being hurt but a) being otherwise threatened, such as having been locked up by the mafia (#26) , or b) hearing about rumors of people in the neighborhood being threatened, such as hearing about people in the neighborhood being injured (#211). Four participants were excluded from the originally 256 participants because of not speaking Italian (*n* = 1), not answering the open fields seriously (*n* = 2), and failing the reading check (*n* = 1) (a reading check was provided where participants had to write “party [festa]” in order to pass the reading check).

### Study design

Ethical approval for the research was granted by the University of Salzburg as part of the FWF P 27457 project. Additionally, participants actively gave their informed consent to participate in the study at the beginning of the survey. All methods were performed in accordance with the relevant guidelines and regulations. An online survey (Limesurvey) in the Italian language was sent out via social networks and via MTurk [There was no difference in the participants ‘answers when controlling for this variable as well as no time differences with regard to completing the survey. Participants recruited via Mturk received 1 Euro for their participation]. The survey can be found under the OSF link provided (see Data Availability Statement). The research was promoted as an online study investigating feelings and perceptions with regard to institutions. The survey started with agreeing to an informed consent, followed by BIS and BAS affective state measures. Then, the mafia threat was introduced and participants were asked about their perception of the mafia. Next, BIS and BAS affective states were measured again. After this, questions on defense mechanisms were asked—again followed by BIS and BAS affective states measures. In the end, demographic data, questions about participants’ Covid-19-related fears (as data was collected in 2021), and the possibility to leave a comment were collected. On the thank-you page, participants were debriefed.

### Measures

#### Mafia manipulation

To make the DT threat salient, participants reflected on the mafia in Italy with regard to four open fields in a way of how a threat has been manipulated as a common practice in threat research (e.g.^50^): (1) How prevalent is the Mafia in Italy? (2) What do you think about the Mafia in Italy? (3) Take two minutes to imagine a situation in which you receive a threat from the mob. Then, take the time to write down what you imagined in this situation. Explain the situation in as much detail as possible. (4) Take time to imagine how that situation would make you feel. Then, take time to describe your feelings and emotions related to that event. (translated version).

#### Manipulation check: DT threat

A first manipulation check was the qualitative analysis of the open questions on the mafia to see whether it was perceived as a threat. To specifically address the DT aspect of the threat, we adapted the twelve items of the *Dirty Dozen Scale* by Jonason and Webster^76^ to the mafia context (e.g., our item: “In my opinion, the Mafiosi are used to expect special favors from others”; original item: “I tend to expect special favors from others “; see Supplementary Appendix 3), as this measure is invariant when assessing DT personality traits in diverse clinical as well as non-clinical groups^77^. As we needed a selective answer (would they categorize mafia as DT yes or no), we used yes or no as answer options instead of a Likert scale. This way, we can better categorize the mafia as either a DT threat or not (see similar procedures, e.g.^78^). The scale had an acceptable internal consistency of α = 0.69.

#### BIS and BAS

The BIS scale consisted of four items, namely *inhibited, worried, restless,* and *insecure.* The first three items have been already used by other scholars (e.g.^79,80^; see Supplementary Appendix 4) to measure BIS. As a perceived threat can go along with feelings of insecurity which can also be understood as a higher activation of the behavioral inhibition system (e.g.^81^), we further decided to add the item “insecure” to our scale (α = 0.91–0.93). To assess BAS, we used the items *relaxed* and *cooperative* by Greenaway et al.^82^ for measuring low approach orientation as well as an adapted version for measuring high approach orientation based on Greenaway et al.^82^ and Reiss et al.^80^ (α = 0.85-0.90; see Supplementary Appendix 4). Thus, six items were used for BAS (*powerful, capable, goal-oriented, determined, relaxed*, *cooperative*). Based on Greenway et al.^82^ participants were asked to indicate the extent to which this series of adjectives describe their current feelings from 1 (*fully disagree*) to 10 (*fully agree*). An overall score was computed for BIS and BAS as an average of all four or six items (see correlations in Table 5).

**Table 5:** Correlations across all BIS and BAS scales.

	1	2	3	4	5
1 BIS_*before Threat*_	1				
2 BIS_*after Threat*_	0.63	1			
3 BIS_*after Defenses*_	0.62	0.89	1		
4 BAS_*before Threat*_	− 0.43	− 0.26	− 0.21	1	
5 BAS_*after Threat*_	− 0.25	− 0.39	− 0.28	0.52	1
6 BAS_*after Defenses*_	− 0.24	− 0.34	− 0.32	0.55	0.88

All correlations are significant (*p* < 0.001).

#### Defense mechanism

An open question assessed how participants would act as a result of the mafia threat to activate defense mechanisms: “How do you think you would act as a result of the imagined situation? Take the time to imagine how you would behave as a result of the imagined situation”. The answers were qualitatively inductively analyzed and then categorized into the General Model of Threat and Defense^47^.

#### Possible buffers

In addition, we asked participants about possible buffers for the mafia threat: “Now we would like you to think of some ways to deal with the Mafia in Italy. Describe below all the ways you think the Mafia can be confronted”. The answers were qualitatively inductively analyzed.

#### Additional measure: Covid-19-related fears

For research outside the present study, we further collected data on participants’ Covid-19-related fears using the MAC-FR scale by Schimmenti et al.^83^ (α = 0.77). Participants were asked to share their sense of agreement from 1 (*fully disagree*) to 5 (*fully agree*) to seven statements, such as “I am frightened about my body being in contact with objects contaminated by the coronavirus”. As this measure is out of the scope of the present research questions, it was not considered in the following analyses.

### Data analysis

All quantitative data analyses were performed with R^84^ using the packages *afex*^85^, *psych*^86^, and *lm.beta*^87^. For the mediation analyses the macro PROCESS^88^ (model 1) was used. QCAmap was used for the qualitative analysis of the defense open questions. The eight steps of the inductive category development according to Mayring^89^ were conducted: (1) Two inter-coders were introduced to our research questions and information on the theoretical background of the research topic was given. (2) The selection criteria, the category definitions, and the level of abstraction (low) were explained to them. (3) The coders worked through the open answers given by the participants and extracted categories from them. (4) After working through 10% of the text materials a revision was made. (5) Afterwards, the text materials were completely worked through. (6) Main categories were created with regard to the Anxiety-to-Approach model; (7) then, an inter-coder agreement check followed. The coders’ results were compared and adjusted. To ensure high reliability, a subcategory was coded only if both coders agreed. (8) In the last step, a review of the final results, the calculation of the frequencies of the extracted categories, and the interpretation of the data followed.

### Ethical approval

The study was included in the FWF project FWF P 27457 and all studies in this project were approved by the Ethics Committee of the University of Salzburg.

## Supplementary Information

Supplementary Information.

## Data Availability

The data that supports the findings of this study are openly available to the reviewers in Open Science Framework under the following link: https://osf.io/rpqyc/?view_only=179ac1ba65f34d9e8ba30a40c117d677. This data has not been published elsewhere but can be made openly available after publishing this manuscript to allow further research at 10.17605/OSF.IO/RPQYC.
