# Supplementary material for: Unveiling the influence of the Italian mafia as a Dark Triad threat on individuals’ affective states and the power of defense mechanisms
Source: Sci Rep. 2023 Jul 25;13:11986. doi: 10.1038/s41598-023-38597-6 (PMC10368718; doi:10.1038/s41598-023-38597-6)
Supplement: Supplementary file 1 — Supplementary Information. [file 41598_2023_38597_MOESM1_ESM.docx]

**Appendices**

**Appendix 1**

*Demographics of the sample*

| Demographics | Percentage of the sample |
| --- | --- |
| Gender | 104 (41 %) male, 148 (59 %) female |
| Nationality | 145 (97 %) Italy, 7 (3 %) Other |
| Residence | 127 (50 %) in South Italy, 70 (28 %) in North Italy, and 55 (22 %) in Central Italy |
| Italian Language | 225 (89 %) Italian as mother tongue, 21 (8 %) as fluent speakers, 6 (2 %) good speakers |
| Education | 113 (45 %) high school diploma, 62 (25 %) bachelor degree, 65 (26 %) master degree, 7 (3 %) before High School, 5 (2 %) doctoral degree |
| Profession | 83 (33 %) workers, 62 (25 %) students, 42 (17.00 %) unemployed, 38 (15 %) freelancers, 21 (8 %) other jobs (e.g. #14 “retired”, #253 “intern”), 6 (2 %) no answer |
| Business owners | 14 (6 %) business owners, 238 (94 %) not |
| Prison experience | 23 (9 %) have been to prison themselves or had friends or family members who have been, 229 (91 %) were not and knew nobody |
| Contact to Mafia | 25 (10 %) friends or family members involved with the mafia, 227 (90 %) no friends or family members involved with the mafia |
| Hurt by the Mafia | 2 % themselves have been hurt by the mafia, 2% friends or family have been hurt by the mafia, 1% friends or family have been killed by the mafia, 8 % other |

**Appendix 2**

*Descriptives and correlations*

| Variable | *M* | *SD* | (1) | (2) | (3) | (4) | (5) | (6) |
| --- | --- | --- | --- | --- | --- | --- | --- | --- |
| (1) Age | 38.22 | 12.79 |  |  |  |  |  |  |
| (2) Gender ^a^ | - | - | .067 |  |  |  |  |  |
| (3) Nationality ^b^ | - | - | -.107^*^ | -.093 |  |  |  |  |
| (4) Italian language | 4.87 | 0.40 | -.088 | .069 | .245^***^ |  |  |  |
| (5) Business owners ^c^ | - | - | .004 | .078 | .064 | -.080 |  |  |
| (6) Prison experience ^d^ | - | - | .108^*^ | .099 | -.054 | -.036 | -.017 |  |
| (7) Contact to mafia ^e^ | - | - | .153^**^ | .046 | -.057 | -.109 | -.023 | .263^***^ |

*Note.* The variables *gender*, *nationality*, *business owners*, *prison experience*, and *contact to mafia* are dummy-coded.

^a^ 0 = male, 1 = female. ^b^ 0 = non-Italian, 1 = Italian. ^c^ 0 = business owner, 1 = no business owner. ^d^ 0 = prison experiences, 1 = no prision experiences. ^e^ 0 = contact to mafia, 1 = no contact to mafia.

^***^*p* < .001. ^**^ *p* < .01. ^*^ *p* < .05.

**Appendix 3**

DT statements based on the Dirty Dozen Scale

- Take time to imagine how that situation would make you feel. Then, take time to describe your feelings and emotions related to that event. [Prenditi il tempo per immaginare in che modo quella situazione ti farebbe sentire. Poi, prenditi il tempo per descrivere le tue sensazioni e le emozioni legate a quell'avvenimento.]
- In my opinion, the Mafiosi [Secondo me, i mafiosi] ...
- are used to want others to look up to them [sono soliti volere che gli altri li ammirino]
- are used to want others to give them attention [sono soliti volere che gli altri gli diano attenzioni]
- are used to expect special favors from others [sono soliti aspettarsi favori speciali dagli altri]
- are used to seek prestige and social success [sono soliti cercare prestigio e successo sociale]
- are used to feel no remorse [sono soliti non provare rimorso]
- are used to be callous and ruthless [sono soliti essere insensibili e spietati]
- are used to not care about the morality or propriety of their actions [sono soliti non preoccuparsi della moralità o della moralità delle loro azioni]
- are used to be cynical [sono soliti essere cinici]
- are used to cheat and lie to achieve their goals [sono soliti truffare e mentire per raggiungere i loro scopi]
- are used to manipulate others to achieve their own ends [sono soliti manipolare gli altri per raggiungere i propri scopi]
- are used to flatter to achieve their own ends [sono soliti lusingare per raggiungere i propri scopi]
- are used to exploit others for their own ends [sono soliti sfruttare gli altri per i propri fini]

**Original: Dirty Dozen Scale** (Jonason and Webster, 2010)

| Machiavellianism | - I tend to manipulate others to get my way - I have used deceit or lied to get my way - I have used flattery to get my way - I tend to exploit others towards my own end |
| --- | --- |
| Psychopathy | - I tend to lack remorse - I tend to be unconcerned with the morality of my actions - I tend to be callous or insensitive - I tend to be cynical |
| Narcissism | - I tend to want others to admire me - I tend to want others to pay attention to me - I tend to seek prestige or status - I tend to expect special favors from others |

**Appendix 4**

To our knowledge, a comprehensive measure to assess BIS and BAS has been missing over the last years and researchers often combined a set of different adjectives instead . Following this approach, we combined different items that have been already used in previous research to measure BIS and BAS. For future research, it should be considered that Franchina et al. (2023) are working on a validated measure to assess BIS and BAS via such adjectives.

**Table A4**

*Measures of BIS and BAS*

|  | **Original** | **Italian translation** | **Original source** | **Note** |
| --- | --- | --- | --- | --- |
| BAS | powerful | potente | Greenway et al. (2015) |  |
|  | c apable/ competent | competente | Greenway et al. (2015)/ Reiss et al. (2020) |  |
|  | competitive |  | Greenway et al. (2015) | This variable was not measured as it is considered as irrelevant for this study’s setting. |
|  | energized |  | Greenway et al. (2015) | This variable was not measured in this study due to technical problems. |
|  | goal-oriented | orientato verso l’obiettivo | Reiss et al. (2020) | These variables were measured due to their relevance in work contexts. |
|  | determined | determinato | Reiss et al. (2020) |  |
| BIS | Inhibited | inibito | Agroskin et al. (2016); Reiss et al. (2020) |  |
|  | worried | preoccupato | Agroskin et al. (2016); Reiss et al. (2020) |  |
|  | anxious |  | Agroskin et al. (2016); Reiss et al. (2020) | These variables were not measured in this study due to technical problems. |
|  | nervous |  | Agroskin et al. (2016); Reiss et al. (2020) |  |
|  | restless | irrequieto | Reiss et al. (2020) |  |
|  | insecure | insicuro |  | This variable was added as threats can often go along with feelings of insecurity (e.g., Long et al., 2022). |
